# Volume versus surface-based cortical thickness measurements: A comparative study with healthy controls and multiple sclerosis patients

**DOI:** 10.1371/journal.pone.0179590

**Published:** 2017-07-06

**Authors:** R. Righart, P. Schmidt, R. Dahnke, V. Biberacher, A. Beer, D. Buck, B. Hemmer, J. S. Kirschke, C. Zimmer, C. Gaser, M. Mühlau

**Affiliations:** 1 Department of Neurology, Klinikum rechts der Isar, Technische Universität München, Munich, Germany; 2 TUM—Neuroimaging Center, Klinikum rechts der Isar, Technische Universität München, Munich, Germany; 3 Department of Statistics, Ludwig-Maximilians-University München, Munich, Germany; 4 Department of Psychiatry and Department of Neurology, Jena University Hospital, Jena, Germany; 5 Munich Cluster for Systems Neurology (SyNergy), Munich, Germany; 6 Department of Neuroradiology, Klinikum rechts der Isar, Technische Universität München, Munich, Germany; Charite Universitatsmedizin Berlin, GERMANY

## Abstract

The cerebral cortex is a highly folded outer layer of grey matter tissue that plays a key role in cognitive functions. In part, alterations of the cortex during development and disease can be captured by measuring the cortical thickness across the whole brain. Available software tools differ with regard to labor intensity and computational demands. In this study, we compared the computational anatomy toolbox (CAT), a recently proposed volume-based tool, with the well-established surface-based tool FreeSurfer. We observed that overall thickness measures were highly inter-correlated, although thickness estimates were systematically lower in CAT than in FreeSurfer. Comparison of multiple sclerosis (MS) patients with age-matched healthy control subjects showed highly comparable clusters of MS-related thinning for both methods. Likewise, both methods yielded comparable clusters of age-related cortical thinning, although correlations between age and average cortical thickness were stronger for FreeSurfer. Our data suggest that, for the analysis of cortical thickness, the volume-based CAT tool can be regarded a considerable alternative to the well-established surface-based FreeSurfer tool.

## Introduction

The cerebral cortex plays a crucial role in cognitive development and decline. Numerous studies have shown that cortical thickness is one of the most important parameters that is related with cognitive functions such as executive functions [1], memory [2], and visual recognition [3]. In addition, various studies have pointed towards a role of cortical thinning as a reliable index of atrophy in neurodegenerative and neurological disease [4].

Measures of cortical thickness have been automated by the use of algorithms. FreeSurfer [5] has been over a decade one of the major software packages for surface-based thickness analyses. FreeSurfer has a large user community and extensive documentation available, with diverse possibilities for advanced pre-processing, such as fixing intensity normalization through control points, removal of dura from the cortical pial surface, as well as statistical modelling, such as vertex-specific general linear model (GLM), or region of interest and atlas-based analyses. It should however be noted that processing time is relatively long (which can amount to 24 hours per individual subject). Therefore the required computational resources can be considerable, particularly if one needs to process a large number of subjects, as is often the case in patient studies. For some beginner users without programming experience, FreeSurfer may have a steep learning curve. In these cases, presenting alternatives that allow for faster processing and rapid learning by the user while at the same time maintaining a high quality would be invaluable. The Computational Anatomy Toolbox (CAT), a toolbox under SPM12, may provide such an alternative. Among other options, CAT provides cortical thickness analyses. This volume-based approach uses a projection-based thickness (PBT) method [6]. Spherical and brain phantoms have confirmed that CAT accurately measures cortical thickness [6]. The pipeline takes about one hour of processing time per subject.

The aim of the current work was to validate CAT compared to FreeSurfer in the context of data derived from a large cohort. Since numerous studies have been published on multiple sclerosis (MS) related cortical thinning [7–11], and on age-related cortical thinning [12–14], we compared the two methods by analyzing a large group of MS patients and healthy controls.

First, we compared the average and standard deviation (SD) of cortical thickness across subjects for CAT and FreeSurfer. Further, we correlated overall estimates of cortical thickness between CAT and FreeSurfer to examine if both methods show a strong correlation. Second, we compared MS patients to healthy controls and expected that similar regions would show significant MS-related thinning for both CAT and FreeSurfer. Third, we compared age-related thinning in MS patients for CAT and FreeSurfer.

## Materials and methods

### Subjects

The group of healthy controls comprised 80 subjects and the MS group 168 subjects. One control participant and two MS patients were removed because CAT had problems estimating the central surface. Thus 79 subjects for the control group (67% female, mean Age = 30.9 yrs) and 166 subjects for the MS group (68% female, mean Age = 30.9 yrs) were analyzed. Patients had a mean disease duration of 3.5 years, and median Expanded Disability Status Scale (EDSS) = 1 (range, 0–3.5). Average lesion volume was 3.8 ml (*SD* = 6.2) and average lesion count was 17.12 (*SD* = 11.3).

The study had been approved by the Ethics Committee of the Medical Faculty, Technical University of Munich. Written informed consent was obtained from healthy participants to undergo MRI scanning for scientific purposes in the context of other imaging studies and from patients to provide their MRI scans, acquired in routine clinical practice, for scientific studies. All subjects were recruited at the same centre (Klinikum Rechts der Isar, Technische Universität München, Germany).

### Acquisition of MR images

All subjects underwent MR scanning at a 3T scanner (Philips Achieva) using the same protocol. We acquired a 3D gradient echo T1w sequence using magnetization-prepared 180 degrees radiofrequency pulses and rapid gradient-echo sampling with a spatial resolution of 1.0 x 1.0 x 1.0 mm^3^, a repetition time (TR) of 9 ms, and an echo time (TE) of 4 ms. For the segmentation of WM lesions, we also acquired a 3D FLAIR sequence with a spatial resolution of 1.0 x 1.0 x 1.5 mm^3^, a TR of 10^4^ ms, a TE of 140 ms, and a time to inversion of 2750 ms.

### Preprocessing

Before preprocessing with either software, we filled white-matter lesions of T1w images by the lesion segmentation tool, version 1.2.3. [15], which is freely available (www.statistical-modeling.de/lst.html). CAT12 Beta version r720 and FreeSurfer version 5.3.0. were both run on the same Linux Workstation. Both software tools are freely available at http://surfer.nmr.mgh.harvard.edu and http://dbm.neuro.uni-jena.de/cat/. The estimation of cortical thickness in CAT is based on the PBT method and is fully automated [6]. It uses tissue segmentation to estimate the WM distance, then projects the local maxima (which is equal to the cortical thickness) to other GM voxels by using a neighbor relationship described by the WM distance. The PBT method allows the handling of partial volume information, sulcal blurring, and sulcal asymmetries. The surface pipeline uses topology correction, spherical mapping, estimation of local surface complexity and local gyrification.

FreeSurfer is semi-automated to construct surface models and estimate amongst other measures the cortical thickness [5]. Surface-based analyses in FreeSurfer involves the removal of non-brain tissue using a hybrid watershed algorithm, automated Talairach transformation, segmentation of subcortical white matter and cortical gray matter, intensity normalization, tessellation of gray/white-matter boundary, automated correction of topological defects and surface deformation to form the gray- and white matter boundary [16]. Cortical thickness was determined as the difference between the pial and white-matter surface [17].

For both CAT and FreeSurfer smoothing kernels of 15 mm were used prior to estimation of vertex specific GLM. Vertices in the medial wall were removed for CAT and FreeSurfer. CAT computation time including pre-processing and surface analysis for an individual subject is about 1 hour. For FreeSurfer the minimal processing time for a subject was 9.5 hours and the maximal time was 23 hours.

### Statistical analyses

We first computed the average and SD of thickness maps in both CAT and FreeSurfer. Whole cortex average thickness was compared between methods using the *t*-test. For between-group analyses we used Welch´s t-test for unequal variances.

In addition, we analyzed the correlation between average thickness values for CAT and FreeSurfer. To facilitate comparison between the two methods, the individual CAT and FreeSurfer thickness values were mapped to the *fsaverage* subject provided by FreeSurfer. For each individual hemisphere, 163842 vertices (i.e., measurement points) were imported into R statistical computing package [18]. Due to different surface registration methods [19], we correlated whole average thickness values and further compared regional effects between the methods using a standard parcellation atlas provided by FreeSurfer [20].

Group differences between MS and healthy controls were analyzed for CAT and FreeSurfer using vertex-specific GLM analyses. In addition to the main effects of group, the interaction between group x method was examined.

Finally, age-related cortical thinning was analyzed for CAT and compared with FreeSurfer, using a linear model. The linear model provided estimates for the slope of age-related cortical thickness alterations. In addition to the age effects for methods separately, we analyzed the interaction between method x age. We applied a statistical threshold of *p*<0.001 (uncorrected) for all vertex-wise analyses.

## Results

### Surface maps of cortical thickness

**Fig 1:**
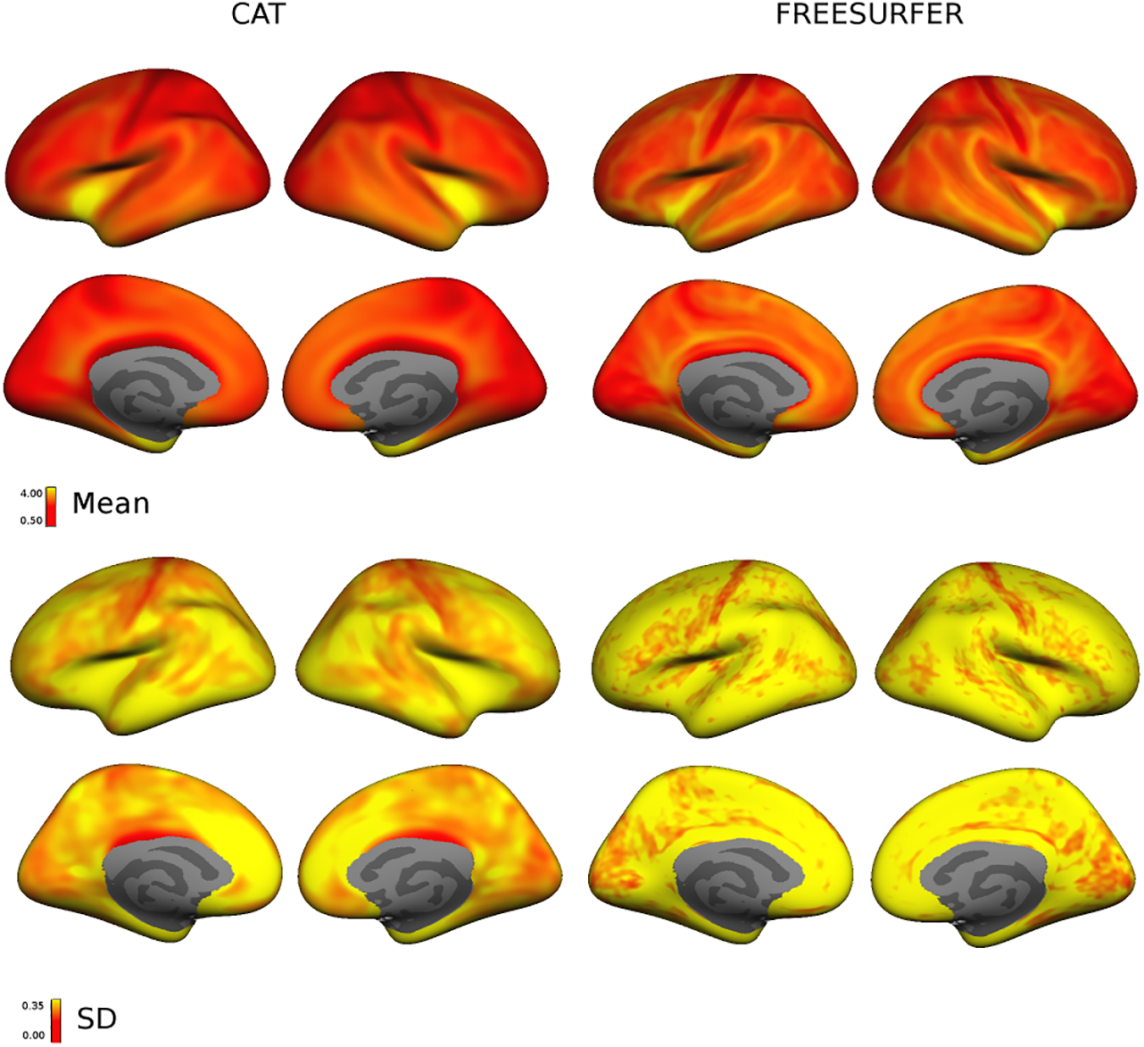
Surface maps of mean and SD of thickness in healthy participants. Maps of mean thickness (row 1–2) and SD (row 3–4) show very similar distributions for FreeSurfer and CAT.

**Fig 2:**
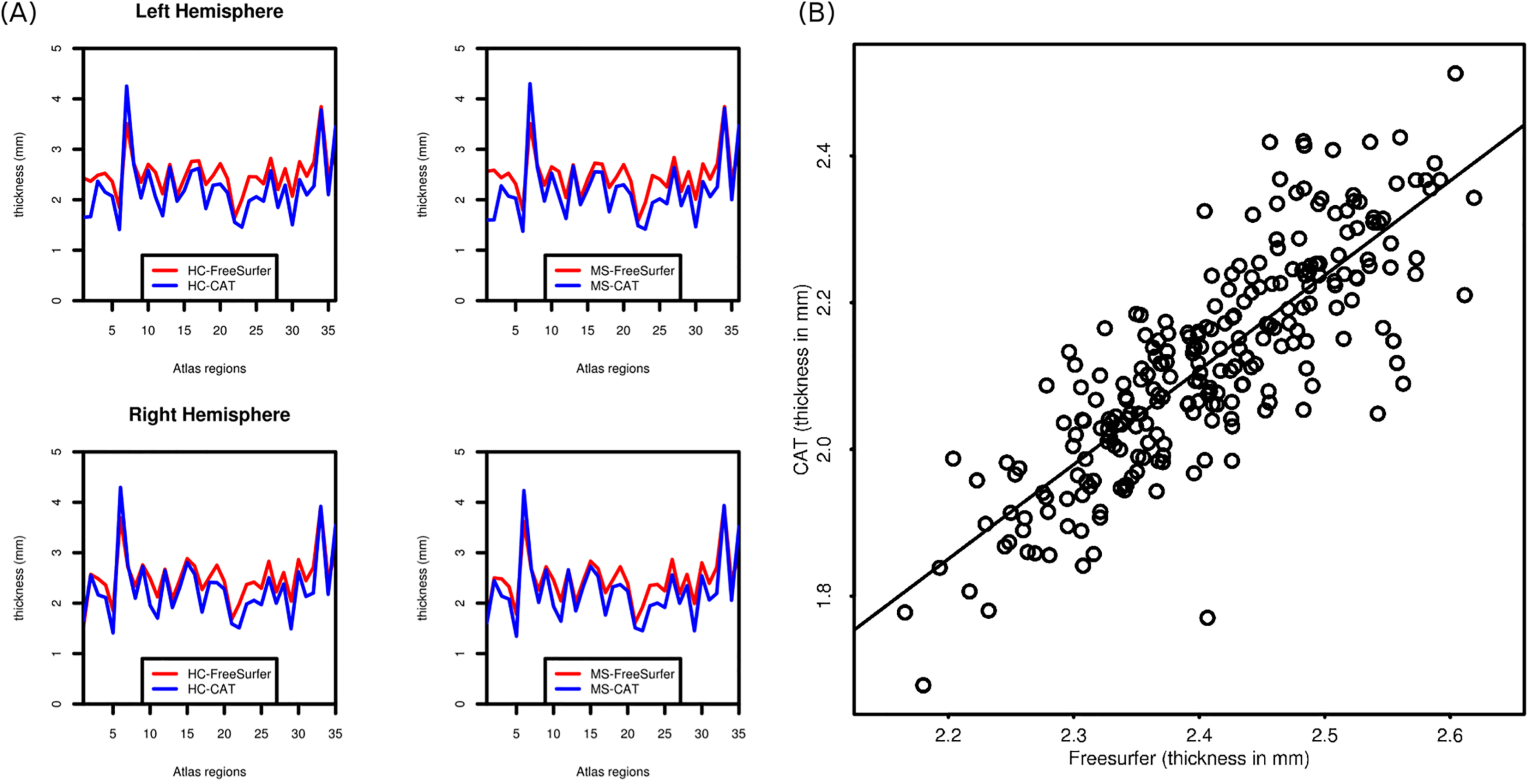
**(a). Thickness values across regions (b) correlation between methods.** Thickness across regions was measured using the parcellation atlas provided by FreeSurfer (Desikan et al., 2006). Numbers on the x-axis are the following labels: 1. Bank of STS, 2. Caudal anterior cingulate cortex, 3. Caudal middle frontal cortex, 4. Cuneus, 5. Entorhinal cortex, 6. Frontal pole, 7. Fusiform cortex, 8. Inferior parietal cortex, 9. Inferior temporal cortex, 10. Insula, 11. Isthmus cingulate cortex, 12. Lateral occipital cortex, 13. Lateral orbitofrontal cortex, 14. Lingual cortex, 15. Medial orbitofrontal cortex, 16. Middle temporal cortex, 17.Paracentral lobule, 18. Parahippocampal gyrus, 19–21: Inferior frontal gyrus: 19. Parsopercularis, 20. Parsorbitalis, 21. Parstriangularis, 22. Pericalcarine cortex, 23. Postcentral gyrus, 24. Posterior cingulate cortex, 25. Precentral gyrus, 26. Precuneus, 27. Rostral anterior cingulate, 28. Rostral middle frontal cortex, 29. Superior frontal cortex, 30. Superior parietal cortex, 31. Superior temporal cortex, 32. Supramarginal gyrus, 33. Temporal pole, 34. Transverse temporal cortex.

The surface map of cortical thickness showed the expected distribution for both methods. The cortex was thinner in the visual areas, whereas the temporal and motor areas were thicker. Maps of the SD showed a similar pattern but more variance in the insular region **(Fig 1).** Using the standard parcellation atlas, cortical thickness showed a highly comparable distribution for CAT and FreeSurfer **(Fig 2A)**.

Average thickness values were higher in FreeSurfer than CAT **(Table 1)**. This was the case for both MS patients, *t =* 20.534, 95% CI: [0.24, 0.29], *df* = 278.22, *p<*0.001 and healthy participants, *t* = 13.041, 95% CI: [0.23, 0.31], *df =* 127.05, *p*<0.001.

**Table 1:** Mean thickness across hemispheres for CAT and FreeSurfer. LH = Left hemisphere, RH = Right hemisphere.

	*Mean thickness in mm (SD)*		*P-values*	*Correlation with age R values P values*	*Correlation with age* *R values* *P values*
	*CAT*	*FreeSurfer*	*CAT vs FreeSurfer*	*CAT*	*FreeSurfer*
LH–Healthy controls	2.127 (0.710)	2.426 (0.603)	*P*<0.001	*-0*.*254*, *P<0*.*05*	*-0*.*401*
LH–MS patients	2.097 (0.716)	2.382 (0.619)	*P*<0.001	*-0*.*195*, *P<0*.*05*	*-0*.*350*, *P<0*.*001*
RH–Healthy Controls	2.180 (0.725)	2.425 (0.612)	*P*<0.001	*-0*.*286*, *P<0*.*05*	*-0*.*446*, *P<0*.*001*
RH–MS patients	2.132 (0.720)	2.379 (0.621)	*P*<0.001	*-0*.*220*, *P<0*.*01*	*-0*.*352*, *P<0*.*001*

We analyzed the correlation between CAT and FreeSurfer for the MS patients. There was a significant correlation between region-wise cortical thickness in CAT and FreeSurfer, with *r* = 0.84, *p*<0.001, 95% CI: [0.79, 0.88] **(Fig 2B)**. For vertex-wise correlation, we obtained a similar value, *r =* 0.89, *p*<0.001.

### Surface-based group comparison

Comparing individual average thickness values of MS patients and healthy participants by t-tests showed no significant differences with CAT, *t* = 1.87, CI:[0.00, 0.08], *df* = 138.28, *p* = 0.0632, but significant differences with FreeSurfer, *t* = 3.51, CI:[0.02, 0.07], *df* = 145.46, *p*<0.001.

**Fig 3:**
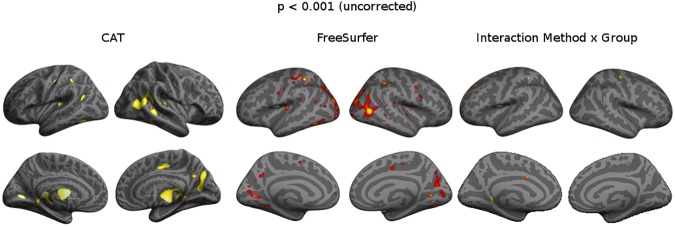
Group differences MS vs HC, p<0.001 (uncorrected). Surface-based statistical maps showing clusters of significant cortical thinning in MS.

Vertex-wise group comparisons yielded similar patterns with both methods. Accordingly, we did not find a meaningful interaction between group and method **(Fig 3)**.

### Effects of age

We further compared CAT and FreeSurfer with regard to age-related cortical thinning of MS patients **(Table 1)**.

**Fig 4:**
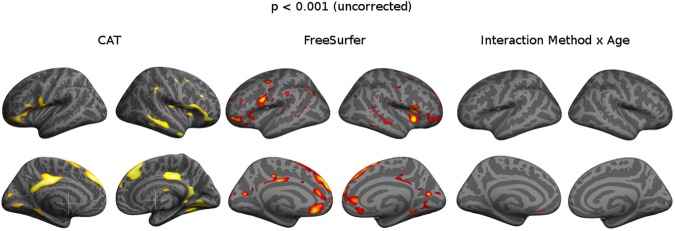
Age-related thinning, p<0.001 (uncorrected). Surface-based statistical maps showing significant clusters of age-related alterations in cortical thickness.

**Fig 5:**
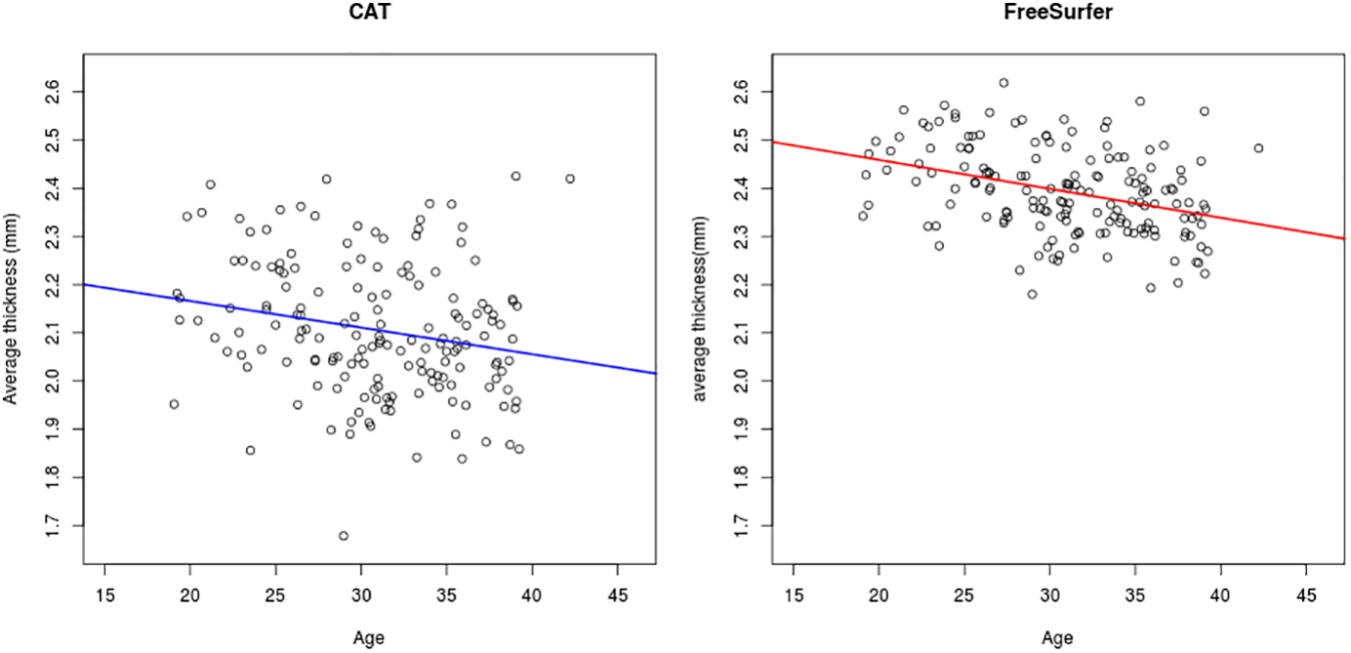
Age-related thinning for CAT and FreeSurfer.

For CAT, there were significant correlations between age and cortical thickness (*r* = -0.21, 95% CI: [-0.06, -0.35], *p* = 0.00645). For FreeSurfer, correlations were stronger (*r* = -0.36, 95% CI: [-0.22, -0.48, *p*<0.001) **(Fig 4)**. A linear model showed that the slope of age-related decline was for CAT -0.0055 and for FreeSurfer -0.0060 **(Fig 5)**.

Vertex-based GLM of the relation between age and thickness showed comparable region-specific effects for CAT and FreeSurfer, with widespread thinning in the superior medial frontal cortex, lateral inferior frontal cortex, supramarginal cortex, lateral temporal cortex, and cingulate cortex. Accordingly, interaction analysis between method and age did not yield significant clusters **(Fig 4)**.

## Discussion

We compared CAT with FreeSurfer. CAT is a VBM-based method to estimate the cortical surface and measure cortical thickness. This study showed that measures of CAT were highly comparable to FreeSurfer with a few exceptions. Overall thickness measures were lower in CAT than FreeSurfer but surface maps of thickness showed similar regional distributions between the two methods. Cortical thickness correlated significantly between the two methods. Comparison of MS patients with an age-matched healthy control group showed highly comparable clusters of MS-related cortical thinning for both methods as also indicated by the lack of a significant interaction between group and method. Finally, vertex-wise validation of well-known age-related cortical thinning showed similar results for CAT and FreeSurfer, notwithstanding that for FreeSurfer correlations of averaged cortical thickness with age were stronger. In sum, taking FreeSurfer as a “gold standard” that has been thoroughly validated by different post-mortem data [17, 21, 22], the current study shows that CAT is capable of estimating cortical thickness in healthy populations and neurological populations such as MS.

The general difference in cortical thickness can depend on the tissue/boundary classification, but also the thickness definition itself, where different approaches are well known to result in varying results [23]. FreeSurfer used the average nearest neighbor metric [17], whereas the mapping scheme of the PBT approach of CAT not only adapts for blurred sulci, but also for blurred gyri. As a result, the thickness of the crones of gyri is much more similar compared to sulcal areas, what results in a more similar cortical pattern.

Alternative methods to estimate the cortical surface may proof to be important for hospitals or research institutes that do not have sufficient computational and staff resources available and/or when processing time would be extensive for a substantial number of patients, for example datasets containing hundreds or thousands of patients. In the case of MS, cortical thickness has been an invaluable measure as a marker of cortical atrophy. It has been discovered that cortical thickness is related with lesion volume on the one hand, and clinical symptoms, cognitive deficits and disability on the other hand [7, 8, 24]. Cortical thickness may be a promising neural marker for clinical trials [25], and the development of fast and reliable software tools to estimate cortical thickness is therefore crucial.

The age-related effect of cortical thinning has been widely reported in healthy participants [12–14], but also in patient populations, such as MS [10] and hereditary small vessel disease [26]. Age is therefore a simple but evident variable to validate the quality of cortical thickness data. We analyzed age-related effects in MS patients because it represented the largest group and therefore there were clearer effects at pre-defined thresholds of *p*<0.001. We found for CAT *β* = -0.005523 and for FreeSurfer *β* = -0.006005. This equals roughly to a decline of 0.05–0.06 mm per 10 years. This estimate in MS patients is slightly higher than the age-related decline of 0.04 mm reported for patients with hereditary small vessel disease [26], and more than twice the reported decline of 0.016 mm per decade in healthy subjects [13]. The aforementioned studies including our own study used cross-sectional designs. Age-associated cortical thinning was widespread but also showed distinctive regional effects, most markedly in lateral temporal areas, superior frontal areas, lateral frontal areas, medial visual areas and the posterior cingulate area. A similar regional pattern of age-related thinning was obtained for CAT and FreeSurfer, even though there was more variance in the CAT measures. Application of FreeSurfer-based masking to select vertices to be included for calculation of the global values in CAT did not change this result. The observed areas of age-related thinning do correspond with regions that were reported for age-related thinning in a large meta study that included 6 different samples totaling 883 subjects [12]. It should nonetheless be mentioned here that FreeSurfer seems more sensitive to capture averaged cortical thinning with age.

The group differences between MS patients and age-matched healthy participants showed highly comparable regional specific thinning for both methods, particularly occupying the parietal cortex, medial occipital cortex and mid-cingulate cortex. Corresponding cortical regions have been reported as well by previous studies [7, 10, 11]. It should be noted that, unlike other studies, we did not observe thinning in the anterior temporal areas [7]. One reason may be that we investigated early stage MS patients, whereas cortical thinning of the temporal poles is particularly observed at longer disease durations [7]. Another interesting observation was cortical thinning in the occipital areas. This is consistent with some previous work [10, 24], though inconsistent with some other studies [7]. A key explanation may be differences in sample sizes, as a low sample size reduces statistical power.

In conclusion, the current work shows that CAT appears a reliable alternative for cortical thickness measures, though both methods show also some differences. CAT may be an option if computational resources are limited while nevertheless sufficiently rapid neuroimaging applications are needed, such as clinical trials where cortical thickness is used as a neural marker. Though softwares such as FreeSurfer likely remain a mainstay for detailed analyses of cortical morphology, an application as CAT may be very important for research scientist or clinicians who do neither have the computing resources in place to analyze their large patient datasets nor the time available to invest in learning and using code-mediated analytical tools. At the moment, CAT however appears less sensitive for detecting differences in averaged cortical thickness.

## References

[1] Schmidt EL, Burge W, Visscher KM, Ross LA. Cortical thickness in frontoparietal and cingulo-opercular networks predicts executive function performance in older adults. Neuropsychology. 2016;30(3):322–31. Epub 2015/10/16. doi: 10.1037/neu0000242 ; PubMed Central PMCID: PMCPmc4767555. 26460586 10.1037/neu0000242 PMC4767555

[2] Engvig A, Fjell AM, Westlye LT, Moberget T, Sundseth O, Larsen VA, et al Effects of memory training on cortical thickness in the elderly. NeuroImage. 2010;52(4):1667–76. Epub 2010/06/29. doi: 10.1016/j.neuroimage.2010.05.041 . 20580844 10.1016/j.neuroimage.2010.05.041

[3] McGugin RW, Van Gulick AE, Gauthier I. Cortical Thickness in Fusiform Face Area Predicts Face and Object Recognition Performance. Journal of cognitive neuroscience. 2016;28(2):282–94. Epub 2015/10/07. doi: 10.1162/jocn_a_00891 . 26439272 10.1162/jocn_a_00891 PMC5034353

[4] Dickerson BC, Bakkour A, Salat DH, Feczko E, Pacheco J, Greve DN, et al The cortical signature of Alzheimer's disease: regionally specific cortical thinning relates to symptom severity in very mild to mild AD dementia and is detectable in asymptomatic amyloid-positive individuals. Cerebral cortex (New York, NY: 1991). 2009;19(3):497–510. Epub 2008/07/18. doi: 10.1093/cercor/bhn113 ; PubMed Central PMCID: PMCPmc2638813. 18632739 10.1093/cercor/bhn113 PMC2638813

[5] Fischl B. FreeSurfer. NeuroImage. 2012;62(2):774–81. Epub 2012/01/18. doi: 10.1016/j.neuroimage.2012.01.021 ; PubMed Central PMCID: PMCPmc3685476. 22248573 10.1016/j.neuroimage.2012.01.021 PMC3685476

[6] Dahnke R, Yotter RA, Gaser C. Cortical thickness and central surface estimation. NeuroImage. 2013;65:336–48. Epub 2012/10/09. doi: 10.1016/j.neuroimage.2012.09.050 . 23041529 10.1016/j.neuroimage.2012.09.050

[7] Sailer M, Fischl B, Salat D, Tempelmann C, Schonfeld MA, Busa E, et al Focal thinning of the cerebral cortex in multiple sclerosis. Brain: a journal of neurology. 2003;126(Pt 8):1734–44. Epub 2003/06/14. doi: 10.1093/brain/awg175 . 12805100 10.1093/brain/awg175

[8] Charil A, Dagher A, Lerch JP, Zijdenbos AP, Worsley KJ, Evans AC. Focal cortical atrophy in multiple sclerosis: relation to lesion load and disability. NeuroImage. 2007;34(2):509–17. Epub 2006/11/23. doi: 10.1016/j.neuroimage.2006.10.006 . 17112743 10.1016/j.neuroimage.2006.10.006

[9] Magon S, Gaetano L, Chakravarty MM, Lerch JP, Naegelin Y, Stippich C, et al White matter lesion filling improves the accuracy of cortical thickness measurements in multiple sclerosis patients: a longitudinal study. BMC neuroscience. 2014;15:106 Epub 2014/09/10. doi: 10.1186/1471-2202-15-106 ; PubMed Central PMCID: PMCPmc4164794. 25200127 10.1186/1471-2202-15-106 PMC4164794

[10] Narayana PA, Govindarajan KA, Goel P, Datta S, Lincoln JA, Cofield SS, et al Regional cortical thickness in relapsing remitting multiple sclerosis: A multi-center study. NeuroImage Clinical. 2012;2:120–31. Epub 2012/01/01. doi: 10.1016/j.nicl.2012.11.009 ; PubMed Central PMCID: PMCPmc3777814. 24179765 10.1016/j.nicl.2012.11.009 PMC3777814

[11] Geisseler O, Pflugshaupt T, Bezzola L, Reuter K, Weller D, Schuknecht B, et al Cortical thinning in the anterior cingulate cortex predicts multiple sclerosis patients' fluency performance in a lateralised manner. NeuroImage Clinical. 2016;10:89–95. Epub 2016/01/14. doi: 10.1016/j.nicl.2015.11.008 ; PubMed Central PMCID: PMCPmc4683425. 26759784 10.1016/j.nicl.2015.11.008 PMC4683425

[12] Fjell AM, Westlye LT, Amlien I, Espeseth T, Reinvang I, Raz N, et al High consistency of regional cortical thinning in aging across multiple samples. Cerebral cortex (New York, NY: 1991). 2009;19(9):2001–12. Epub 2009/01/20. doi: 10.1093/cercor/bhn232 ; PubMed Central PMCID: PMCPmc2733683. 19150922 10.1093/cercor/bhn232 PMC2733683

[13] Salat DH, Buckner RL, Snyder AZ, Greve DN, Desikan RS, Busa E, et al Thinning of the cerebral cortex in aging. Cerebral cortex (New York, NY: 1991). 2004;14(7):721–30. Epub 2004/04/01. doi: 10.1093/cercor/bhh032 . 15054051 10.1093/cercor/bhh032

[14] Hutton C, Draganski B, Ashburner J, Weiskopf N. A comparison between voxel-based cortical thickness and voxel-based morphometry in normal aging. NeuroImage. 2009;48(2):371–80. Epub 2009/06/30. doi: 10.1016/j.neuroimage.2009.06.043 ; PubMed Central PMCID: PMCPmc2741580. 19559801 10.1016/j.neuroimage.2009.06.043 PMC2741580

[15] Schmidt P, Gaser C, Arsic M, Buck D, Forschler A, Berthele A, et al An automated tool for detection of FLAIR-hyperintense white-matter lesions in Multiple Sclerosis. NeuroImage. 2012;59(4):3774–83. doi: 10.1016/j.neuroimage.2011.11.032 . 22119648 10.1016/j.neuroimage.2011.11.032

[16] Dale AM, Fischl B, Sereno MI. Cortical surface-based analysis. I. Segmentation and surface reconstruction. NeuroImage. 1999;9(2):179–94. Epub 1999/02/05. doi: 10.1006/nimg.1998.0395 . 9931268 10.1006/nimg.1998.0395

[17] Fischl B, Dale AM. Measuring the thickness of the human cerebral cortex from magnetic resonance images. Proceedings of the National Academy of Sciences of the United States of America. 2000;97(20):11050–5. Epub 2000/09/14. doi: 10.1073/pnas.200033797 ; PubMed Central PMCID: PMCPmc27146. 10984517 10.1073/pnas.200033797 PMC27146

[18] RCoreTeam. R: A language and environment for statistical computing. 2016.

[19] Yotter RA, Thompson PM, Gaser C. Algorithms to improve the reparameterization of spherical mappings of brain surface meshes. Journal of neuroimaging: official journal of the American Society of Neuroimaging. 2011;21(2):e134–47. doi: 10.1111/j.1552-6569.2010.00484.x . 20412393 10.1111/j.1552-6569.2010.00484.x

[20] Desikan RS, Segonne F, Fischl B, Quinn BT, Dickerson BC, Blacker D, et al An automated labeling system for subdividing the human cerebral cortex on MRI scans into gyral based regions of interest. NeuroImage. 2006;31(3):968–80. Epub 2006/03/15. doi: 10.1016/j.neuroimage.2006.01.021 . 16530430 10.1016/j.neuroimage.2006.01.021

[21] Rosas HD, Liu AK, Hersch S, Glessner M, Ferrante RJ, Salat DH, et al Regional and progressive thinning of the cortical ribbon in Huntington's disease. Neurology. 2002;58(5):695–701. Epub 2002/03/13. . 11889230 10.1212/wnl.58.5.695

[22] Popescu V, Klaver R, Versteeg A, Voorn P, Twisk JW, Barkhof F, et al Postmortem validation of MRI cortical volume measurements in MS. Human brain mapping. 2016;37(6):2223–33. doi: 10.1002/hbm.23168 . 26945922 10.1002/hbm.23168 PMC6867379

[23] Lerch JP, Evans AC. Cortical thickness analysis examined through power analysis and a population simulation. NeuroImage. 2005;24(1):163–73. doi: 10.1016/j.neuroimage.2004.07.045 . 15588607 10.1016/j.neuroimage.2004.07.045

[24] Calabrese M, Atzori M, Bernardi V, Morra A, Romualdi C, Rinaldi L, et al Cortical atrophy is relevant in multiple sclerosis at clinical onset. Journal of neurology. 2007;254(9):1212–20. Epub 2007/03/16. doi: 10.1007/s00415-006-0503-6 . 17361339 10.1007/s00415-006-0503-6

[25] Ontaneda D, Fox RJ, Chataway J. Clinical trials in progressive multiple sclerosis: lessons learned and future perspectives. The Lancet Neurology. 2015;14(2):208–23. Epub 2015/03/17. doi: 10.1016/S1474-4422(14)70264-9 ; PubMed Central PMCID: PMCPmc4361791. 25772899 10.1016/S1474-4422(14)70264-9 PMC4361791

[26] Righart R, Duering M, Gonik M, Jouvent E, Reyes S, Herve D, et al Impact of regional cortical and subcortical changes on processing speed in cerebral small vessel disease. NeuroImage Clinical. 2013;2:854–61. Epub 2013/11/02. doi: 10.1016/j.nicl.2013.06.006 ; PubMed Central PMCID: PMCPmc3777834. 24179837 10.1016/j.nicl.2013.06.006 PMC3777834

